# A worldwide perspective on clinical characteristics and treatment of youth with monogenic diabetes in the SWEET registry

**DOI:** 10.1210/jendso/bvag064

**Published:** 2026-03-23

**Authors:** Lily Deng, Stefanie Lanzinger, Kristina Casteels, Fred Cavallo, Luisa De Sanctis, Ana Laura Fitas, Carol Huang, Jaehyun Kim, Konstantina Patouni, Stepanka Pruhova, Craig E Taplin, Martin Tauschmann, Mansa Krishnamurthy

**Affiliations:** Division of Endocrinology, Cincinnati Children's Hospital Medical Center, University of Cincinnati College of Medicine, Cincinnati, OH 45229, USA; Institute of Epidemiology and Medical Biometry, CAQM, Ulm University, Ulm, Germany; German Center for Diabetes Research (DZD), Munich-Neuherberg, Germany; Department of Pediatrics, University Hospitals Leuven, Leuven, Belgium; Department of Development and Regeneration, KU Leuven, Leuven, Belgium; Hospital Nacional de Niños, San José, Costa Rica; Pediatric Endocrinology, Regina Margherita Children Hospital–Department of Public Health and Pediatric Sciences, University of Turin, Turin, Italy; Department of Pediatric Endocrinology, Hospital Dona Estefânia, Lisboa, Portugal; Department of Pediatrics and Biochemistry and Molecular Biology, University of Calgary, Calgary T2N 1N4, Canada; Department of Pediatrics, Seoul National University Bundang Hospital, Seongnam, South Korea; Diabetes Center, First Department of Paediatrics, “P&A Kyriakou” Children’s Hospital, Athens, Greece; Department of Pediatrics, Second Faculty of Medicine, Charles University in Prague and Motol University Hospital, Prague, Czech Republic; Department of Endocrinology and Diabetes, Perth Children’s Hospital, Perth 6009, Australia; Department of Pediatrics and Adolescent Medicine, Medical University of Vienna, Vienna, Austria; Division of Endocrinology, Cincinnati Children's Hospital Medical Center, University of Cincinnati College of Medicine, Cincinnati, OH 45229, USA

**Keywords:** maturity-onset diabetes of the young, MODY, monogenic diabetes, global diabetes trends, MODY treatment, genetic diabetes, MODY comorbidities

## Abstract

**Aims:**

To characterize youth with monogenic diabetes worldwide in the SWEET database and define trends in clinical care and outcomes.

**Methods:**

Youth with monogenic diabetes between the ages of 0 and 21 years from 44 worldwide centers in the SWEET registry divided into global regions were studied in this retrospective analysis. This included 690 youth with data at diabetes diagnosis and/or follow-up at 1 year and 214 patients with data at both time points. Demographics, comorbidities, and treatments were evaluated.

**Results:**

Globally, mean age and hemoglobin A1c (HbA1c) at diagnosis were 10.1 years (SD 4.57 years) and 6.9% (52 mmol/mol) (SD 1.7%, 18 mmol/mol), respectively. At 1-year follow-up, mean HbA1c decreased by 0.4%. Average body mass index (BMI) at diabetes diagnosis was in the normal range (World Health Organization BMI SD score 0.31). At diabetes diagnosis, 3.6% presented in diabetic ketoacidosis (DKA). Seven percent were treated with oral antidiabetes medications or glucagon-like peptide-1 receptor agonists at diabetes diagnosis, increasing to 15.3% at follow-up. Overall, treatment with insulin increased by 10% at follow-up.

**Conclusion:**

Worldwide, patients with monogenic diabetes typically present during late childhood/early adolescence with mild elevation in HbA1c, normal BMI, and lack of DKA. Regional differences in demographics and treatment modalities highlight heterogeneity in presentation and management of monogenic diabetes, impacting clinical care.

Monogenic diabetes encompasses a variety of genetic forms of diabetes, affecting 1% to 5% of the pediatric population with diabetes [1]. Diagnostic criteria for some subsets of monogenic diabetes [including maturity-onset diabetes of the young (MODY)] include negative islet cell autoantibodies, presentation of diabetes prior to 25 years of age, and pathogenic variants in monogenic diabetes genes on molecular genetic testing [2, 3]. Variants in monogenic diabetes genes lead to pancreatic β-cell dysfunction and are commonly inherited in an autosomal dominant manner [4].

It is crucial to differentiate monogenic diabetes from type 1 or type 2 diabetes, as treatment approaches may vary based on the specific diagnosis. Glucokinase (GCK) diabetes typically does not require the use of medications and can be managed by lifestyle modifications alone [5], while hepatocyte nuclear factor 1 α (HNF1A) diabetes and hepatocyte nuclear factor 4 α (HNF4A) diabetes typically respond well to sulfonylureas and glucagon-like peptide-1 receptor agonist (GLP-1 RA) therapy but may ultimately require insulin therapy [1, 6-9]. Additionally, different forms of monogenic diabetes are associated with a range of risks for complications and involvement of other organ systems. Thus, a timely and specific diagnosis of monogenic diabetes supports optimal clinical management and targeted therapeutic approaches.

The literature regarding monogenic diabetes has predominantly involved White European populations [4]; however, more recent reports have described cohorts with monogenic diabetes in India [10] and Asia [11]. With advances in genetic testing, diagnoses of monogenic diabetes have increased along with further understanding of the pathophysiology underlying its various forms [12, 13]. While the spectrum of monogenic diabetes phenotypes and treatments has been characterized [4, 9, 10, 14, 15], there has not been a comprehensive, worldwide analysis of monogenic diabetes phenotypes and associated treatment patterns in a diverse population.

The aim of this study was to characterize patients with monogenic diabetes across 44 centers in the global SWEET (Better control in Pediatric and Adolescent diabeteS: Working to crEate cEnTers of Reference) database, define trends in clinical care, and report outcomes for these patients. We hypothesized that clinical presentation at diagnosis of diabetes and treatment would differ by region.

## Methods

This retrospective cohort study utilized the SWEET database to evaluate characteristics of patients with monogenic diabetes over a time period of 13 years. SWEET is a global network of centers that provide care for individuals with diabetes. Genes used to identify the diagnosis of monogenic diabetes included those associated with MODY: *HNF4A*, *GCK*, *HNF1A*, pancreatic and duodenal homeobox 1 (*PDX1*), hepatocyte nuclear factor-1 β (*HNF1B*), neurogenic differentiation factor 1 (*NEUROD1*), carboxyl ester lipase (*CEL*), insulin (*INS*), ABCC8 ATP binding cassette subfamily C member 8 (*ABCC8*), and potassium inwardly rectifying channel subfamily J member 11 (*KCNJ11*). Patients with variants in B-lymphoid tyrosine kinase (*BLK*), Krueppel-like factor 11 (*KLF11*), and paired box 4 (*PAX4*) were excluded. Participating centers in the SWEET database establish the diagnosis of monogenic diabetes according to their local protocols and subsequently submit only the confirmed diagnosis, with the option to include the implicated gene, to the central database [16]. All centers use the same monogenic diabetes gene diagnostic codes when submitting data. Patients under the age of 21 years with data from an initial diagnosis of diabetes and/or at 1-year follow-up between 2010 and 2023 were included in this study. All centers with reported data of interest for this study were included. Global regions were defined based on regions within the SWEET database: Asia/Middle East, Australia/New Zealand, Europe, North America, and Central/South America. Asia/Middle East included data from 3 centers (Republic of Korea, Thailand, and Turkey); 2 centers represented Australia/New Zealand; and the European region encompassed 29 centers from Germany, England, Portugal, Netherlands, Sweden, Czech Republic, Poland, Luxembourg, Greece, France, Croatia, Belgium, Italy, Spain, Lithuania, Austria, Croatia, Bulgaria, and Serbia. The data from 8 centers in the United States and Canada comprised the North American region, and the 2 centers in Central/South America were Costa Rica and Chile.

SWEET started as a project from 2008 to 2011 to develop centers of reference for pediatric and adolescent diabetes services across the European Union with the goal to improve quality and reduce inequalities in diabetes care. It has since expanded to become a global network of centers that provide care for individuals with diabetes. Each participating center collects local data through electronic health records, clinical databases, existing registries, or the standardized SWEET DPV documentation software and then submits pseudonymized data to the SWEET database at the Institute of Epidemiology and Medical Biometry in Ulm, Germany, biannually. The data team at Ulm University validates this data and adds it to a common database [17, 18].

Patient demographics including age, sex, hemoglobin A1c (HbA1c), body mass index (BMI) SD score per the World Health Organization [19], presence of diabetic ketoacidosis (DKA) at the time of diagnosis, and comorbidity screenings for autoimmune thyroid disease and celiac disease were evaluated for each region of interest. To adjust for differences between laboratories, the multiple of the mean method was used to mathematically standardize HbA1c values to the reference range of the Diabetes Control and Complications Trial [4-6% (21-43 mmol/mol)] [20]. Patients with monogenic diabetes were identified by physician-reported diabetes classification by each center in data sent to SWEET. Monogenic diabetes gene distribution by region was compared. Treatment with insulin versus oral antidiabetes (OAD) medications, GLP-1 RAs, and combination therapy with OAD and insulin therapy was evaluated for each region of interest. Use of continuous glucose monitor (CGM) or insulin pump was compared between each region.

### Statistics

Unadjusted comparisons between regions were conducted using the Wilcoxon test for continuous outcomes and chi-squared test for binary variables. Continuous outcomes (HbA1c and BMI-SDS) were analyzed using linear regression adjusting for sex, current age, and age at diabetes onset and treatment. Binary outcomes (insulin treatment, OAD/GLP-1-RA treatment) were investigated using logistic regression models adjusted for sex, current age, and age at diabetes onset. All analyses were conducted using SAS version 9.4 (TS1M7, SAS Institute Inc, Cary, NC). GraphPad Prism 10.1.2 (GraphPad Software, Inc., San Diego, CA) was used for data presentation. A *P*-value of <.05 was considered statistically significant.

## Results

### Genetic characteristics of the patient population

Information on monogenic diabetes types among 44 centers worldwide was extracted from the SWEET database. A total of 115 146 patients with diabetes were identified in the SWEET database. Within this population, 1462 patients were diagnosed with monogenic diabetes (MODY); 690 had data at the time of diabetes diagnosis, of which 214 patients had data at both diagnosis and 1-year follow-up. Within this cohort, GCK diabetes was the most common form of monogenic diabetes, comprising 59% of the total population with monogenic diabetes, followed by HNF1A diabetes at 18% and HNF1B diabetes at 7% (Fig. S1 [21]). Similarly, within each geographical region of interest, GCK diabetes had the highest prevalence except for in North America (Fig. S2 [21]). Other common monogenic diabetes variants among each geographical region included *HNF1B* and *HNF1A*. Rarer gene variants associated with monogenic diabetes, *INS* and *KCNJ11,* were also found within our cohort. There were 276 patients diagnosed with “genetic defects of the β cell: others” without a further genetic diagnosis of monogenic diabetes type. These were excluded from analyses of patient demographics across regions. Within SWEET, Europe had the highest reported number of youth/adolescents with monogenic diabetes (n = 438, 63.5%), followed by North America (n = 98, 14.2%), Central/South America (n = 72, 10.4%), Australia (n = 45, 6.5%), and Asia/Middle East (n = 37, 5.4%).

When adjusted for sex, current age, age at diabetes onset, and treatment type, average HbA1c at time of diabetes diagnosis was highest in INS diabetes at 11.2% followed by HNF1B diabetes at 9.6%. Adjusted BMI was highest in INS diabetes (SDS 2.48) followed by HNF4A (SDS 1.01) (Table S1 [21]). At follow-up, the greatest adjusted change in average HbA1c was seen in INS diabetes (−5.5%), followed by HNF1B diabetes (−3.4%), and KCNJ11 diabetes (−3.3%) (Table S1 [21]).

### Demographics of youth/adolescents with monogenic diabetes at time of diabetes diagnosis and follow-up

Demographics of youth/adolescents with monogenic diabetes in the global cohort were evaluated at the time of diabetes diagnosis and 1-year follow-up. At the diagnosis of diabetes, the average age was 10.13 years with an average HbA1c of 6.9% (52 mmol/mol) (Table 1), and 3.6% of patients with monogenic diabetes presented in DKA at the time of diabetes diagnosis. Average World Health Organization BMI SDS was 0.31 at diabetes diagnosis. There was an equal distribution of females (50.2%) in the cohort to males (49.8%).

**Table 1:** General characteristics of patients with monogenic diabetes across all regions of the world at diagnosis of monogenic diabetes

	Percent	Mean ± SD
Age of diabetes diagnosis (years)		10.13 ± 4.57
BMI at diabetes diagnosis (WHO BMI SDS)		0.31 ± 1.27
HbA1c at diabetes diagnosis (%)		6.94 ± 1.67
HbA1c at diabetes diagnosis (mmol/mol)		52 ± 18
Sex (%)		
Female	50.2	
Male	49.8	
Ketoacidosis at diagnosis (%)		
Present	3.6	
Absent	96.4	
Percentage of diabetic ketoacidosis within each monogenic diabetes type at diagnosis (%)		
*ABCC8*	22.2	
*CEL*	50	
*GCK*	1.2	
*HNF1B*	6.9	
*INS*	11.8	
*KCNJ11*	38.5	

Total n = 414. Patients labeled as “other” diabetes were excluded from calculations.

Abbreviations: BMI, body mass index; HbA1c, hemoglobin A1c; SDS, SD score; WHO, World Health Organization.

**Figure 1:**
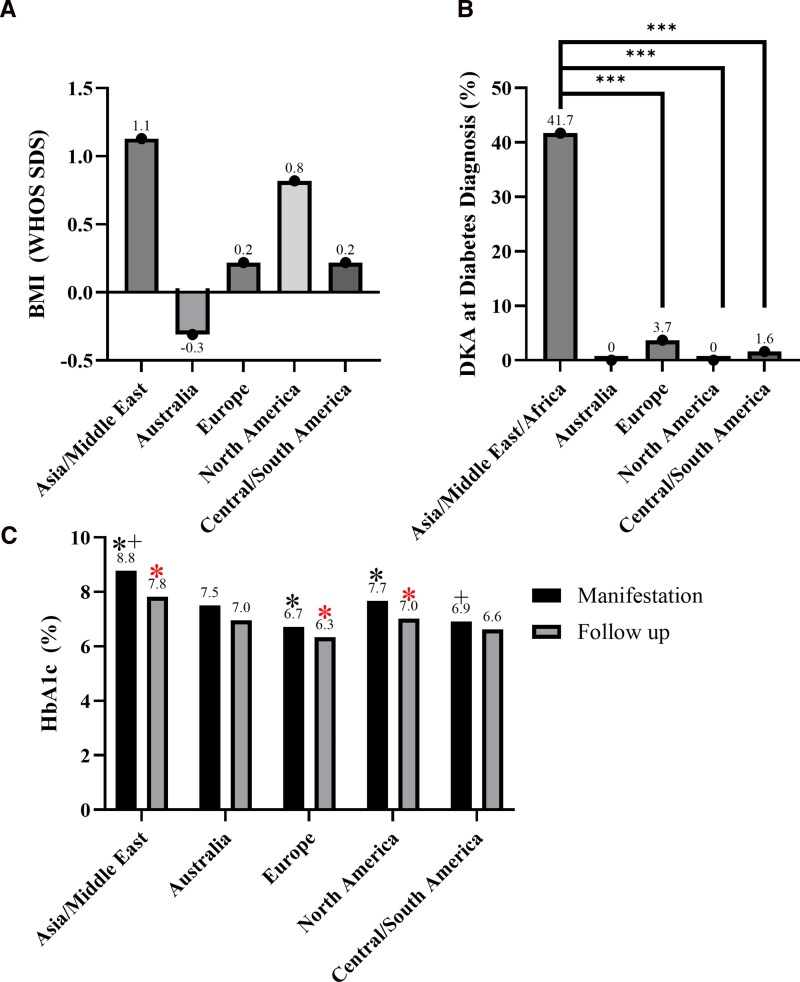
Demographics of the cohort with monogenic diabetes by each geographical region. (A) WHO BMI SDS by each geographical region. The highest average BMI SDS was seen in Asia/Middle East compared to the lowest in Australia. (B) Percentage of patients with monogenic diabetes in each geographic region of interest who presented in DKA at initial diagnosis of diabetes. A significantly higher percentage of patients in Asia/Middle East presented in DKA compared to Europe and Central/South America. ***P-value <.001. (C) Average HbA1c at diagnosis of diabetes and follow-up in 1 year by geographic region. HbA1c improved at follow-up for patients in all regions. Individuals from European centers had significantly lower HbA1c than those from Asia/Middle East and North America at diagnosis (*P-value < .005 and ^+^*P*-value < .05) and *follow-up (*P*-value < .05). Abbreviations: BMI, body mass index; DKA, diabetic ketoacidosis; HbA1c, hemoglobin A1c; SDS, SD score; WHO, World Health Organization.

Demographics for youth/adolescents with monogenic diabetes were compared between geographical regions of interest. Youth/adolescents in Central/South America were diagnosed with diabetes earlier in life at an average of 8.9 years of age compared to those in Europe (10.1 years), Australia (11.0 years), North America (11.0 years), and Asia/Middle East (12.4 years). Between each region, there was no significant difference in BMI SDS, but the highest average BMI SDS was in Asia/Middle East (1.1) with the lowest in Australia (−0.3) (Fig. 1A). Among the monogenic diabetes types, DKA at diabetes diagnosis was seen in 22.2% (n = 2) of all youth with ABCC8 diabetes, 50% (n = 1) in CEL diabetes, 1.2% (n = 3) in GCK diabetes, 6.9% (n = 2) in HNF1B diabetes, 38.5% (n = 5) in KCNJ11 diabetes, and 11.8% (n = 2) in INS diabetes. Individuals with monogenic diabetes in Asia/Middle East had a significantly higher rate of DKA at diagnosis (41.7%) compared to those in Europe (3.7%) and Central/South America (1.6%) (Fig. 1B). Average HbA1c at diabetes diagnosis was highest in Asia/Middle East (8.8%, 72 mmol/mol) with the lowest average HbA1c in Europe (6.7%, 50 mmol/mol).

For the total population with monogenic diabetes across all regions, average HbA1c at 1-year follow-up improved to 6.6% (49 mmol/mol). All regions showed improved HbA1c from diagnosis to 1-year follow-up with the greatest improvement in average HbA1c seen in individuals from Asia/Middle East (−1%, −10 mmol/mol). Individuals from European centers had the lowest average HbA1c at follow-up (6.3%, 52 mmol/mol) followed by those from centers in Central/South America (6.6%, 49 mmol/mol), Australia (7.0%, 52 mmol/mol), North America (7.0%, 53 mmol/mol), and then Asia/Middle East (7.8%, 62 mmol/mol%), although youth/adolescents in Asia/Middle East had the largest average improvement in HbA1c at follow-up compared to HbA1c at diagnosis (Fig. 1C).

The prevalence of comorbidities such as hypertension and dyslipidemia, along with other autoimmune diseases, were evaluated by geographical region. Across all regions, average systolic blood pressure was in the normal range. Average low-density lipoprotein cholesterol (LDL-C) was below 100 mg/dL in all regions except Australia (112.14 mg/dL). A higher number of youth/adolescents were screened for dyslipidemia via LDL-C in Europe (n = 68), with rarer LDL-C screening in the remaining regions of Asia/Middle East (n = 4), Central/South America (n = 5), Australia (n = 1), and North America (n = 1). Within each region, the greatest percentage of youth/adolescents with monogenic diabetes screened for thyroid hormone dysfunction was reported in Asia/Middle East (58.3%) compared to Europe (36.4%), North America (26.7%), Central/South America (22.6%), and Australia (18.2%). In terms of autoimmune thyroid disease, 36.4% in Europe were screened for thyroid disease with 7.0% positive for antithyroglobulin antibodies among those screened, 10.0% positive for antithyroid peroxidase antibodies among those screened, and 1.5% diagnosed with autoimmune hypothyroidism among all screened for thyroid disease. Other regions did not have reported data on diagnoses of autoimmune hypothyroidism. Moreover, 15.7% of all youth/adolescents were screened for celiac disease, and among all regions, no youth/adolescents who were screened at diabetes diagnosis had positive antibodies for celiac disease, but 4.4% of youths/adolescents with monogenic diabetes in North America and 1.1% in Europe had been previously diagnosed with celiac disease.

### Treatment modalities

**Figure 2:**
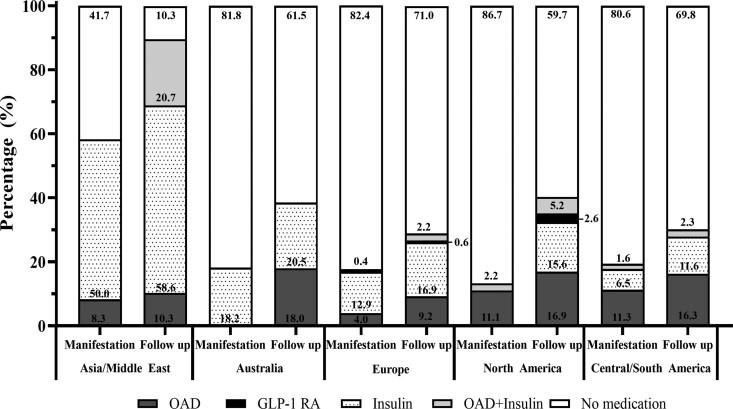
OAD medication, GLP-1 RA treatment, OAD and insulin treatment, insulin treatment, and no medication at manifestation compared to follow-up across all regions. Usage of OAD medications increased at follow-up for all regions along with use of OAD and insulin combined therapy. Back box = GLP-1 RA, dotted box = insulin therapy, dark gray box = OAD, light gray box = OAD and insulin, white box = no medication. Abbreviations: GLP-1 RA, glucagon-like peptide-1 receptor agonist; OAD, oral antidiabetes.

Treatment at the time of diagnosis was evaluated by region. Across all regions, youth/adolescents in Asia/Middle East had the highest percentage treated with insulin (50%) followed by Australia (18.2%), Europe (12.9%), Central/South America (6.5%), and North America (0%) (Fig. 2). Central/South America had the greatest percentage of youth/adolescents treated with OADs (11.3%) compared to all other regions. At least 80% of youth/adolescents did not require treatment with medication at the initial diagnosis of diabetes, with the exception of youth/adolescents in Asia/Middle East.

At 1-year of follow-up after diagnosis, treatment with OADs significantly increased in the total, global cohort (*P*-value < .05) (Fig. 2). Treatment with insulin at follow-up also increased across all regions (Fig. 2). Use of combination therapy with OAD and insulin increased in all regions except Australia at follow-up. GLP-1 RA use increased in Europe (+0.2%) and North America (+2.6%), but the remaining regions did not have youth/adolescents treated with GLP-1 RAs.

**Figure 3:**
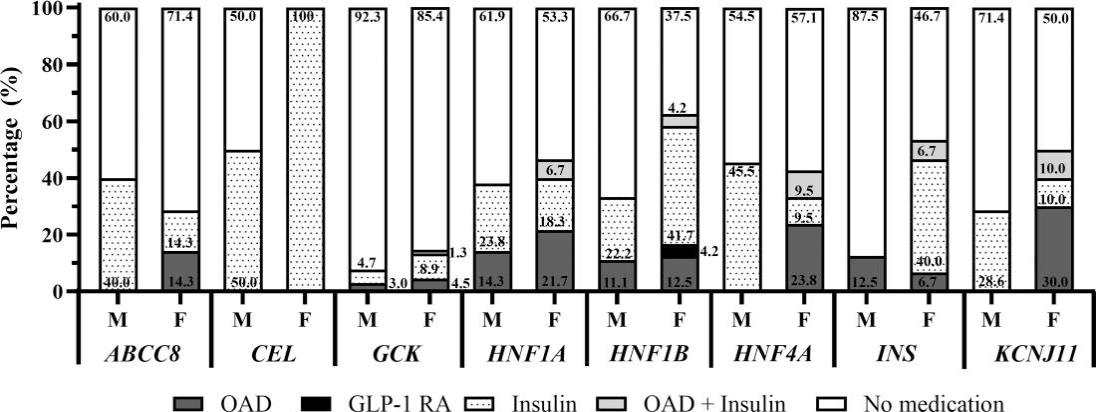
Global percentage of youth/adolescents with monogenic diabetes treated with GLP-1 RA, insulin, OADs, and OAD and insulin combination therapy by monogenic diabetes gene. OAD medication usage increased at follow-up for all monogenic diabetes types except INS diabetes and CEL diabetes. Insulin usage decreased at follow-up in ABCC8 diabetes, HNF4A diabetes, and KCNJ11 diabetes but increased in the remaining monogenic diabetes types. M = manifestation of diabetes, F = follow-up at 1 year after diabetes manifestation. Black box = GLP-1 RA, dotted box = insulin therapy, dark gray box = OAD, light gray box = OAD and insulin, white box = no medication. Abbreviations: ABCC8, ATP-binding cassette subfamily C member 8; CEL, carboxyl ester lipase; GLP-1 RA, glucagon-like peptide-1 receptor agonist; HNF4A, hepatocyte nuclear factor 1 α; INS, insulin; KCNJ11, potassium inwardly rectifying channel subfamily J member 11; OAD, oral antidiabetes.

Across the global cohort, OAD use increased at follow-up for all monogenic diabetes types except CEL diabetes (treatment solely with insulin) and INS diabetes where insulin treatment increased and OAD treatment decreased (Fig. 3). GLP-1 RA was used for treatment of 4.2% of youth with HNF1B diabetes at follow-up. At follow-up, insulin treatment was commonly used in CEL diabetes (100%), HNF1B diabetes (41.7%), and INS diabetes (40%), whereas treatment with OADs at follow-up was more frequent in KCNJ11 diabetes (30.0%), HNF4A diabetes (23.8%), and HNF1A diabetes (21.7%). The percentage of patients who did not require pharmacologic therapy decreased at follow-up across all monogenic diabetes types except in HNF4A diabetes and ABCC8 diabetes. GCK diabetes had the highest percentage of youth/adolescents who did not require pharmacologic treatment at follow-up (85.4%). Additionally, ABCC8 diabetes, HNF1A diabetes, HNF4A diabetes, and KCNJ11 diabetes all had ≥ 50% of patients who did not require pharmacologic treatment at follow-up 1 year after diabetes manifestation.

### Diabetes technology

In addition to treatment with medication, the use of diabetes technology was evaluated globally and by region. Overall, 0.2% of youth/adolescents with monogenic diabetes were started on CGM at diagnosis of diabetes, with the use of CGM increasing by 7 times at the time of follow-up in 1 year. By region, 8.3% of youth/adolescents in Asia/Middle East had CGM initiation at diabetes diagnosis compared to 0% on CGM in all other regions. Furthermore, a higher percentage of youth/adolescents in North America (100%, n = 1) and Asia/Middle East (50%, n = 3) were started on subcutaneous insulin infusions/insulin pumps compared to Europe (22.0%, n = 9), Australia (0%), North America (2.2%, n = 1), and Central/South America (0%) at diabetes diagnosis. At follow-up in 1 year, the greatest percentage of youth/adolescents with monogenic diabetes on CGM was seen in Australia (5.1%, n = 2), followed by Asia/Middle East (3.5%, n = 1) and Europe (1.3%, n = 4). North America and Central/South America did not report any youth/adolescents with monogenic diabetes on CGM at follow-up. The percentage of youth/adolescents with monogenic diabetes using insulin pumps at follow-up increased in Europe (27.5%, n = 19), Asia/Middle East (76%, n = 19), and North America (44.4%, n = 8). Australia had no change in the percentage of youth/adolescents on insulin pumps at follow-up (5.0%, n = 2). Central/South America had no patients on insulin pumps at follow-up.

## Discussion

This multicenter retrospective cohort study utilizing the SWEET database characterizes the global clinical presentation and treatment of patients with monogenic diabetes. A strength of this study is the global perspective it provides for characteristics of monogenic diabetes. Similar to previously reported studies [1, 2], GCK diabetes was 1 of the most common forms of monogenic diabetes seen in the global population and also in each region, with a higher global prevalence also of HNF1A diabetes, HNF1B diabetes, and HNF4A diabetes. Characteristics of patients with monogenic diabetes at initial diabetes presentation in our global cohort were notable for mild elevation in HbA1c, average age of 10 years at diagnosis of diabetes, normal BMI, low rates of DKA at diabetes onset, and low risk of diabetes comorbidities. These characteristics are consistent with prior studies [2, 3]. However, a small percentage of patients in our cohort presented in DKA, which has been reported in large case series in Turkey [22] but otherwise has not been frequently reported. These data show that the presence of DKA at diagnosis should not preclude a diagnosis of monogenic diabetes if the clinical phenotype and family history are suggestive of monogenic diabetes. Thyroid dysfunction and celiac disease, along with dyslipidemia and hypertension, were rare in our cohort. Lower rates of hypertension and dyslipidemia are within expectations given the younger ages within the cohort. In particular, the prevalence of celiac disease in our global cohort with monogenic diabetes (1.2%) was similar to the 0.5% to 1% reported in the general population [23] and less than reported in the population with type 1 diabetes [24]. This is unsurprising given the lack of shared autoimmune risk seen in youth with type 1 diabetes.

There were several notable differences in patient demographics and treatment of monogenic diabetes by geographical region. The highest percentage of youth/adolescents with monogenic diabetes who presented in DKA was seen in Asia/Middle East. Asia/Middle East also had the highest percentage of patients on insulin therapy, perhaps due to the higher percentage of patients who presented in DKA. The standard of care in diabetes involves starting insulin therapy for treatment of type 1 diabetes [25] and for metabolically unstable type 2 diabetes. For patients who present with new-onset diabetes in DKA, the initial differential diagnosis includes type 1 diabetes, which could likely contribute to the larger number of youth/adolescents on insulin therapy at diabetes diagnosis in Asia/Middle East. Furthermore, Asia/Middle East had the highest average age at diabetes diagnosis and had a higher percentage of monogenic diabetes types associated with eventual insulin requirement (such as *INS*, *HNF1A*, *HNF4A*, and *HNF1B*) compared to GCK diabetes, which requires either no treatment or lifestyle modification alone. Delayed diagnosis of monogenic diabetes could contribute to the increased presentation in DKA and continued insulin requirement at follow-up. The total number of youth who presented in DKA at diabetes diagnosis included a majority of monogenic diabetes types associated with eventual insulin requirement (such as *INS*, *HNF1B*, *KCNJ11*, and *ABCC8*) but also included 3 youth with GCK diabetes. This is particularly of interest as DKA is not typically seen with GCK diabetes, but given the limitations of the data reported within the database, the factors associated with the presentation in DKA were unable to be ascertained. This is an area for future studies. While Asia/Middle East had a higher percentage of patients with diabetes technology, this could be due to the continued need for insulin therapy as a higher percentage of patients required insulin at follow-up in Asia/Middle East compared to other regions. North America also had an increase in the percentage of patients on insulin therapy at follow-up and had a similar increase in insulin pump use. However, all regions had an increase in patients treated with OADs, combination OAD and insulin, or GLP-1 RAs at follow-up, likely due to transition to targeted therapy when the etiology of monogenic diabetes was identified. This change of therapy is consistent with prior studies that have reported efficacy of targeted therapy for specific monogenic diabetes types using GLP-1 RAs and OADs, such as sulfonylureas [6, 8, 14]. Furthermore, after diagnosis of monogenic diabetes, targeted therapy approaches with OADs were commonly seen in KCNJ11 diabetes, HNF4A diabetes, and HNF1A diabetes, mirroring findings in existing literature of targeted treatment with sulfonylureas [9]. Interestingly, the percentage of patients with monogenic diabetes who required medication increased at follow-up across all regions. This could potentially be due to the progression of diabetes after initial diagnosis. However, rates of those not requiring therapy were still high at follow-up.

Of note, HbA1c at diabetes presentation was significantly lower in Europe compared to other regions, perhaps due to an earlier diagnosis of monogenic diabetes or a higher percentage of GCK diabetes. Availability of monogenic diabetes genetic testing affects the duration to monogenic diabetes diagnosis and transition to targeted treatment. Potentially, limited availability of monogenic diabetes genetic testing and a higher prevalence of monogenic diabetes types associated with insulin deficiency in Asia/Middle East could contribute to the higher percentage of patients on insulin compared to other regions. The higher percentage of patients on insulin in Asia/Middle East could also be due to clinical care algorithms as patients who present in DKA could potentially be misdiagnosed with type 1b diabetes and thus be treated with long-term INS therapy. This study highlights a need for global availability of cost-efficient monogenic diabetes genetic testing, which would allow timely diagnosis of monogenic diabetes, optimization of clinical patient care, and targeted treatment.

Limitations of this study include its retrospective nature and reliance on reported database information, which may obscure the underlying causes of variations observed in clinical presentations. Furthermore, there were 276 patients diagnosed with “genetic defects of the β cell: others” without a genetic diagnosis of monogenic diabetes type. For this group of individuals, there was no specific monogenic diabetes gene reported by the reporting centers of SWEET. Thus, this category could have contained various forms of monogenic diabetes. Within some of the geographic regions, this represented almost half of the total population, who were given the diagnosis of monogenic diabetes; these patients were not included in analyses of patient demographics between each region. Additionally, some regions were represented by a small number of centers, leading to smaller populations that may not be representative for those geographical regions. Furthermore, each geographical region was defined by location but could also contain heterogeneity in regard to socioeconomic factors, which can impact treatment options and timely diagnosis of monogenic diabetes. Plans for future studies could include a detailed investigation of the types of monogenic diabetes contained within this other category, which would increase the sample size for each region. Finally, monogenic diabetes is an uncommon diagnosis, affecting 1% to 5% of the population with diabetes [1], which presents a challenge for the identification of large patient cohorts. However, our cohort of 690 patients with monogenic diabetes is 1 of the larger cohorts described to date.

## Conclusion

In conclusion, patients with monogenic diabetes across the world typically present during late childhood/early adolescence with mildly elevated HbA1c, normal BMI, low rate of DKA, and lack of diabetes comorbidities at diabetes onset. Varying differences in prevalence of MODY subtypes across geographical regions further highlight the importance of timely diagnosis of monogenic diabetes and its implications for clinical care, including personalized, targeted treatment for patients.

## Data Availability

Some or all datasets generated during and/or analyzed during the current study are not publicly available but are available from the corresponding author on reasonable request.
